# Editorial: Microbial ecology and ecosystems from a Southern perspective

**DOI:** 10.3389/fmicb.2022.1098400

**Published:** 2022-12-05

**Authors:** Verónica Molina, Céline Lavergne, Yoanna Eissler, Pilar Junier

**Affiliations:** ^1^Departamento de Ciencias y Geografía, Observatorio de Ecología Microbiana, Universidad de Playa Ancha, Valparaíso, Chile; ^2^HUB Ambiental UPLA, Universidad de Playa Ancha, Valparaíso, Chile; ^3^Centro de Investigación Oceanográfica COPAS COASTAL, Universidad de Concepción, Concepción, Chile; ^4^Laboratory of Aquatic Environmental Research, Centro de Estudios Avanzados, Universidad de Playa Ancha, Valparaíso, Chile; ^5^Instituto de Química y Bioquímica, Facultad de Ciencias, Universidad de Valparaíso, Valparaíso, Chile; ^6^Laboratory of Microbiology, Institute of Biology, University of Neuchâtel, Neuchâtel, Switzerland

**Keywords:** Antarctic, high altitude, polyextreme environments, microbial diversity, fungi, bacteria, archaea, virus

The Southern Hemisphere, with its geographical and landscape peculiarities, concentrates many hotspots of diverse and extreme environments where microbial life thrives. It includes “pristine” or less anthropogenically impacted environments, such as high-altitude and high-latitude ecosystems, which are highlighted in the content of the Research Topic (word cloud Figure 1). Among the former, the Andes plateau hosts ecosystems that are ideal to study the role of environmental factors in shaping microbial life. Physical factors like continuous mixing regime were studied on the zonation of planktonic microbial communities in high-altitude (>4,400 m above sea level m.a.s.l) polymictic tropical lakes (Aguilar et al.). Physicochemical factors such as extreme changes in conductivity, temperature and nutrients were identified as factors accounting for viruses-to-prokaryotes (VPR) spatial and diel dynamics in Salar de Huasco high-altitude wetland (located at 3,800 m.a.s.l) (Eissler et al.). Similar factors also determined the spatial variability of VPR in other contrasting environments of the Southern Hemisphere, such as marine (Indian, Atlantic, and Pacific Ocean) and Antarctic lakes (Eissler et al.). Also, the salinity gradients typically found in Salar de Huasco and associated with the main lake, ponds, springs, and streams were the natural laboratory selected to survey the diversity of lysis-resistant bacteria and archaea cells in sediments and microbial mats (Corona Ramírez et al.). Salinity is a significant factor that modulates cellular metabolisms. The study of halophilic fungi isolates (Jímenez-Gómez et al.) demonstrates the different molecular mechanisms for resistance in hypersaline systems. Salinity was also a determinant factor leading to the high complexity of microbial communities from semi-arid mangrove soils in Brazil (Tavares et al.).

**Figure 1:**
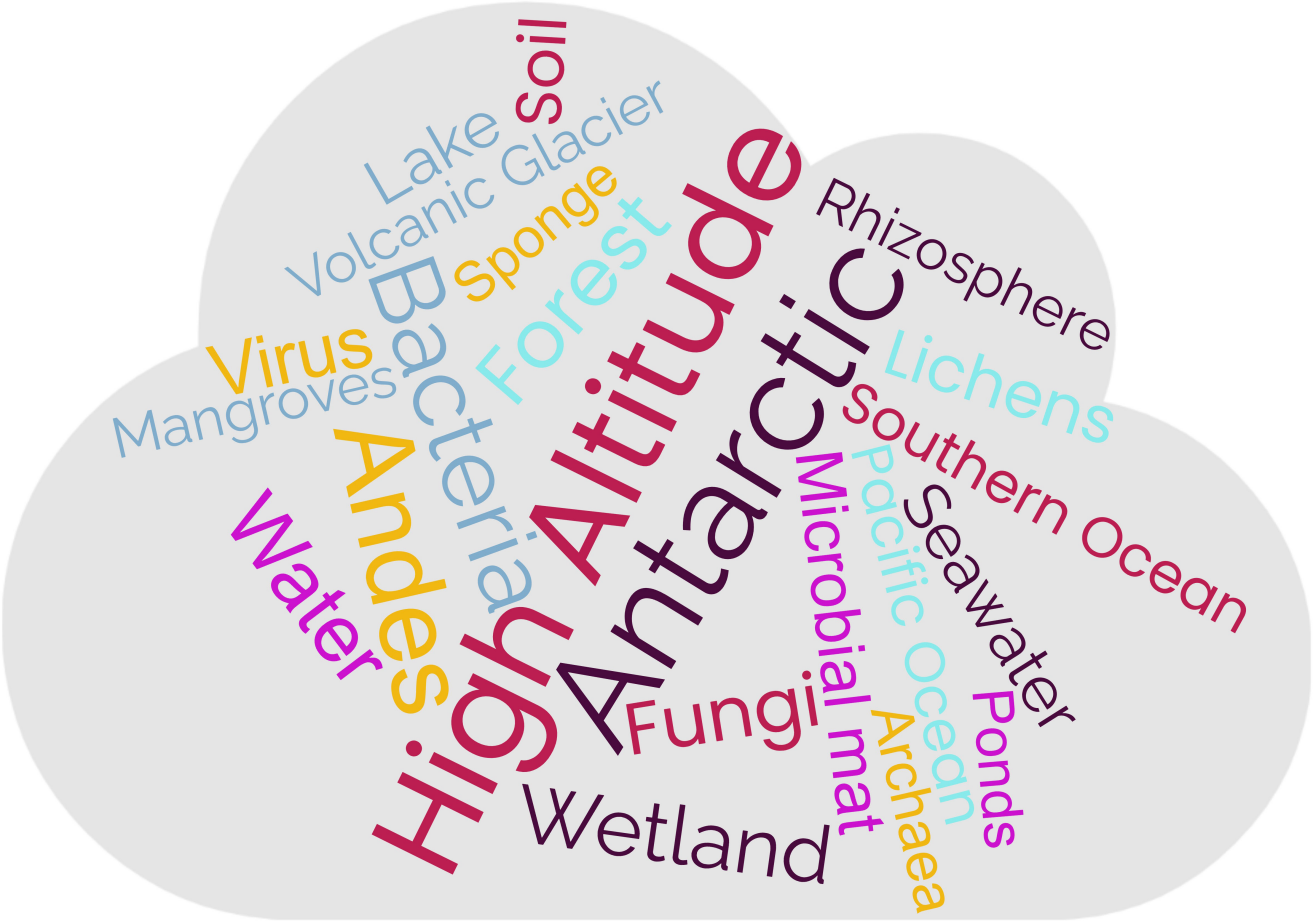
Word cloud showing the proportion of ecosystems and organisms highlighted by the Research Topic.

On the other hand, high-latitude ecosystems such as Patagonia and the Antarctic Peninsula are natural laboratories for investigating the processes determining microbial diversity, their dispersion, activities, and adaptation under changing conditions. In these extreme environments, the association between microorganisms and their hosts is key, as shown in the case of the Antarctic sponge core microbiome which was closely associated with the host species (Cristi et al.), rather than determined by environmental conditions (Happel et al.). Patagonia and the Antarctic Peninsula, still considered pristine, are a reservoir of undescribed microbial species, particularly fungal species (Villanueva et al.). The polyextreme Deception Island in the Antarctic Peninsula where the microbial communities of volcanic glaciers are composed by both psychrophilic and thermophilic microorganisms such as *Psychrobacter* and *Thermogemmatispora*, respectively (Garcia-Lopez et al.), has received particular attention.

The Southern Ocean encompasses several biogeographical provinces and other spatial scale of variability associated with oceanic circulation and fronts, influencing the distribution of microbial populations, acting as “interconnectors” or “ecological boundaries” for microbial populations like *Phaeocystis* (Sow et al.) and *Spirochaeta* (Schwob et al.).

Experimental approaches with complex communities are necessary to unravel ecological responses and interaction of microbial communities under global warming scenarios. For example, studying the tolerance and resistance of key functional microbial groups to temperature, such as the response of benthic methanotrophic Antarctic Lake communities (Roldán et al.). Likewise, changing macronutrient stoichiometry by experimental amendments of phototrophic microbial mats in the Byers Península (Camacho et al.) showed shifts in the microbial communities and their contribution to biogeochemical processes after perturbations.

Functional redundancy could be significant for ecosystem function, especially under the harsh conditions of cold extreme environments. This was shown, for instance, by the monitoring of ammonia oxidation process in the surface sea waters of the West Antarctic Peninsula, where diverse and active nitrifying assemblages were reported to reach high abundances and rates during late-summer (Alcamán-Arias et al.). Macronutrients could explain the microbial changes associated with the wildfires in bulk soil and rhizosphere of a native Sclerophyll Forest in central Chile (Aponte et al.). Different natural perturbations affecting ecosystems, such as hurricanes, cyclones, heat waves, among other extreme events, were predicted to become more common due to climate change with potential significant consequences in nature, including microbial community dynamics. After a tropical cyclone in 2010, the ponds of Pozas Rojas were inundated and homogenized, and then isolated again after several years, representing a natural perturbed situation to study classical niche differentiation questions tested by Garcia-Ulloa et al. for microbial communities. Despite the homogenization effect, this research identified the importance of interspecific interactions compared to environmental control alone accounting for the microbial community establishment in the different ponds (Garcia-Ulloa et al.). Finally, several mechanisms involve the interaction between microorganisms and vegetation including the way in which the microbiome is acquired. Those mechanisms are relevant to understand host health as reported by Muster et al. for phosphate solubilization by lichens in native vs. invasive tree species in the Patagonian Forest, and by Guajardo-Leiva et al. showing differential sites origin for the acquisition of bacteria compared to fungi for rhizosphere establishment by Antarctic vascular plant.

As a general trend, in the Southern Hemisphere, the microbial ecology studies included in this Research Topic were focused mainly in extreme and/or pristine environments using multiple approaches including gold-standard methods for microbial identification of prokaryotic and eukaryotic domains (16S, 18S, 28S rRNA genes and ITS), abundance determinations and functional potential, for example, by profiling metabolic capabilities (Aponte et al.) and by measuring the specific activity rates of microbial processes and meta-omic techniques (Aguilar et al.; Alcamán-Arias et al.; Camacho et al.). Still, there is a need to expand and increase our knowledge of more microbial communities' traits in the Southern Hemisphere ecosystems, using integrative studies to improve and deepen our understanding of their adaptation to extreme and changing environmental conditions. Funding remains a major limitation to increase our baseline knowledge of the South, and in the frame of global change and the effect of anthropogenic perturbations, more than ever, scientific exchange and close collaborations are urgently needed to overcome the lack of funding. Furthermore, the representation of women in research is another major challenge to overcome. Although this is not an exclusive problem of the Southern Hemisphere, an even smaller proportion of women researchers has been found in low-income countries (Quadrio-Curzio et al., 2020), which are mainly located in the South. In this Research Topic 55–60% of first and corresponding authors were women, which is a good sign of change. Thus, financial incentives, collaboration, and inclusiveness would certainly have a positive impact on our knowledge of the wide range of ecosystems driven by microbial processes in the Southern Hemisphere.

## Author contributions

VM, CL, YE, and PJ contributed to conception and design of the study. VM and CL wrote the first draft of the manuscript. YE and PJ wrote sections of the manuscript. All authors contributed to manuscript revision, read, and approved the submitted version.
